# Ruscogenin Attenuates Cerebral Ischemia-Induced Blood-Brain Barrier Dysfunction by Suppressing TXNIP/NLRP3 Inflammasome Activation and the MAPK Pathway

**DOI:** 10.3390/ijms17091418

**Published:** 2016-08-29

**Authors:** Guosheng Cao, Nan Jiang, Yang Hu, Yuanyuan Zhang, Guangyun Wang, Mingzhu Yin, Xiaonan Ma, Kecheng Zhou, Jin Qi, Boyang Yu, Junping Kou

**Affiliations:** 1 Jiangsu Key Laboratory of Traditional Chinese Medicine Evaluation and Translational Research, Department of Complex Prescription of Traditional Chinese Medicine, China Pharmaceutical University, Nanjing 211198, China; caoguosheng2006@163.com (G.C.); nancyjiangnan@163.com (N.J.); huyangzi2014@163.com (Y.H.); cpuzhang27@163.com (Y.Z.); wgyhefei@163.com (G.W.); yinmingzhu2008@126.com (M.Y.); yuefei_86@126.com (K.Z.); yaoyuelingxing@163.com (J.Q.); 2 Cellular and Molecular Biology Center, China Pharmaceutical University, Nanjing 211198, China; maxiaonan512@126.com

**Keywords:** ruscogenin, ischemic stroke, blood-brain barrier, inflammasome, MAPK

## Abstract

Ruscogenin, an important steroid sapogenin derived from *Ophiopogon japonicus*, has been shown to inhibit cerebral ischemic injury. However, its potential molecular action on blood-brain barrier (BBB) dysfunction after stroke remains unclear. This study aimed to investigate the effects of ruscogenin on BBB dysfunction and the underlying mechanisms in middle cerebral artery occlusion/reperfusion (MCAO/R)-injured mice and oxygen–glucose deprivation/reoxygenation (OGD/R)-injured mouse brain microvascular endothelial cells (bEnd.3). The results demonstrated that administration of ruscogenin (10 mg/kg) decreased the brain infarction and edema, improved neurological deficits, increased cerebral brain flow (CBF), ameliorated histopathological damage, reduced evans blue (EB) leakage and upregulated the expression of tight junctions (TJs) in MCAO/R-injured mice. Meanwhile, ruscogenin (0.1–10 µM) treatment increased cell viability and trans-endothelial electrical resistance (TEER) value, decreased sodium fluorescein leakage, and modulated the TJs expression in OGD/R-induced bEnd.3 cells. Moreover, ruscogenin also inhibited the expression of interleukin-1β (IL-1β) and caspase-1, and markedly suppressed the expression of Nucleotide-binding domain (NOD)-like receptor family, pyrin domain containing 3 (NLRP3) and thiredoxin-interactive protein (TXNIP) in vivo and in vitro. Furthermore, ruscogenin decreased reactive oxygen species (ROS) generation and inhibited the mitogen-activated protein kinase (MAPK) pathway in OGD/R-induced bEnd.3 cells. Our findings provide some new insights into its potential application for the prevention and treatment of ischemic stroke.

## 1. Introduction

Ischemic stroke is the leading cause of destructive cerebrovascular disease, which can have serious effects on human health and is characterized by high morbidity, high mortality, and high incidence [1]. To date, the treatment options for ischemic stroke still have several limitations, due to the complexity of the biochemical and molecular mechanisms underlying the disease [2]. Among previous findings, there is increasing evidence indicating that blood-brain barrier (BBB) dysfunction is one of the key pathological mechanisms in ischemic stroke [3]. As a highly specialized endothelial tissue, the function of the BBB is modulated by the cerebral microvascular endothelium, which can prevent peripheral leukocyte recruitment into the brain parenchyma [4]. Many factors are known to lead to BBB breakdown after ischemic stroke. In particular, tight junctions (TJs) such as Zonula occludens-1 (ZO-1) and occludin play key roles in preventing peripheral leukocyte recruitment and mediating of the function of the BBB following ischemic stroke [5]. Thus, TJs might be important target molecules for potential drug design and development.

Previous studies have indicated that inflammation plays a significant role in stroke pathology [6,7,8]. It is a major cause of BBB disruption and leads to secondary injury and dysfunction that increases the risk of bleeding and limits the tissue recovery, which makes it a promising target for intervention in strokes [9,10]. The innate immune system plays an important role in inflammatory cascade; in particular, inflammasome is one of the most important components in the innate immune system and is assembled by pattern recognition receptors into multimeric protein complexes [11]. NLRP3 (Nucleotide-binding domain (NOD)-like receptor family, pyrin domain containing 3) is an inflammasome for which the structure and function have been extensively investigated, and it is activated in the onset of ischemic stroke [12,13,14]. Meanwhile, abundant evidence has confirmed that NLRP3 can regulate the activation of caspase-1 and processes pro-inflammatory cytokines into mature forms of interleukin-1β (IL-1β) and ultimately induces pyroptotic cell death [12,13,15]. Moreover, recent studies have demonstrated that thioredoxin-interactive protein (TXNIP) activation is a key event linked to inflammation; after stroke, it dissociates from the complex and rapidly binds to NLRP3 inflammasome via reactive oxygen species (ROS). Thus, inhibiting the expression of TXNIP could decrease the activation of inflammasome following ischemic stroke [16,17]. In addition, mitogen-activated protein kinase (MAPK) is one of the most important signaling pathways, heavily regulating the BBB disruption and inflammasome activationin in stroke pathology [13,18].

**Figure 1:**
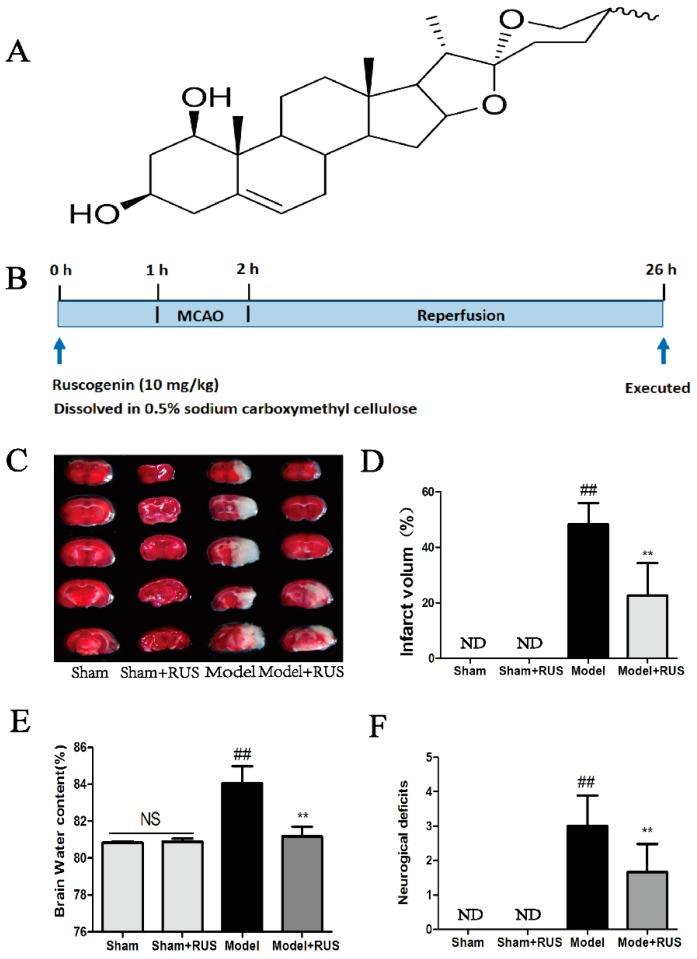
Effects of ruscogeinin on brain tissue injury in mice following MCAO/R. (**A**) The chemical structure of ruscogenin; (**B**) schematic diagram showing the experimental protocol. The experimental groups included the Sham group, Sham + RUS (ruscogenin) group, Model (MCAO/R) group and Model + RUS group. Ruscogenin was administrated intragastrically 1 h before occlusion, and the mice were reperfused after MCAO 1 h and sacrificed after a further 24 h, respectively; (**C**,**D**) representative of TTC-stained brain sections and quantitative analysis of infarct volume in different groups; (**E**,**F**) quantitation of brain water contents and neurological deficits scores in different groups. ND means “not detected”. NS means “not significant”. The data (*n* = 6) are expressed as means ± SD. ^##^ *p* < 0.01 vs. Sham, ** *p* < 0.01 vs. Model.

Ruscogenin (for which the chemical structure shown in Figure 1A) is a major bioactive steroid sapogenin that is found in the roots of *Ophiopogon japonicus* (*Thumb.*) Ker-Gawl., and it has been widely used for the treatment of chronic inflammatory and cardiovascular diseases for many years [19,20]. Pharmacological studies have demonstrated that ruscogenin exerts its beneficial effects on mouse neutrophil activation and pulmonary arterial hypertension, mainly due to its anti-inflammatory and anti-thrombotic activities [21,22]. Furthermore, ruscogenin has been confirmed to reduce cerebral ischemic injury via the nuclear factor-κB (NF-κB)-mediated inflammatory pathway in mice [23]. However, the effects and potential mechanisms of ruscogenin on the BBB dysfunction or inflammasome activation remain to be explored.

Given the key role of NLRP3 inflammasome related to BBB dysfunction in the ischemic stroke pathology [12,14], the present study was conducted to further verify the in vivo effects of ruscogenin on BBB integrity in middle cerebral artery occlusion/reperfusion (MCAO/R)-injured mice, and explore the possible underlying pathways in an in vitro cell model of the BBB using oxygen–glucose deprivation/reoxygenation (OGD/R)-injured mouse brain microvascular endothelial cells (bEnd.3). Our results may provide some new insights into its potential application for treatment of cerebrovascular diseases.

## 2. Results

### 2.1. Ruscogenin Decreased MCAO/R-Induced Brain Infarct Volume and Edema, and Improved Behavioral Outcomes

As shown in Figure 1B, the experimental groups included the Sham group, Sham + RUS (ruscogenin) group, Model (MCAO/R) group and Model + RUS group. Ruscogenin was administrated intragastrically 1 h before occlusion, and the mice were reperfused after MCAO 1 h and sacrificed after a further 24 h, respectively. Infarction volume, brain water content and neurological deficits were detected 24 h after reperfusion. The 2,3,5-triphenyltetrazolium chloride (TTC)-staining image and quantitative analysis of brain infarction indicated that pretreatment with ruscogenin (10 mg/kg) led to smaller infarct size compared to the model group (Figure 1C,D). In addition, the result of brain water content demonstrated that administration with ruscogenin resulted in a marked decrease on brain edema 24 h after reperfusion (Figure 1E). Also, pretreatment with ruscogenin resulted in significant improvement in neurobehavioral deficits compared with the model group (Figure 1F). No obvious change was observed between the Sham and Sham + RUS groups in the above experiments. All data demonstrated that pretreatment with ruscogenin improved the MCAO/R-induced brain tissue injury.

### 2.2. Ruscogenin Ameliorated Histopaological Damage and Cerebral Blood Flow Following MCAO/R

**Figure 2:**
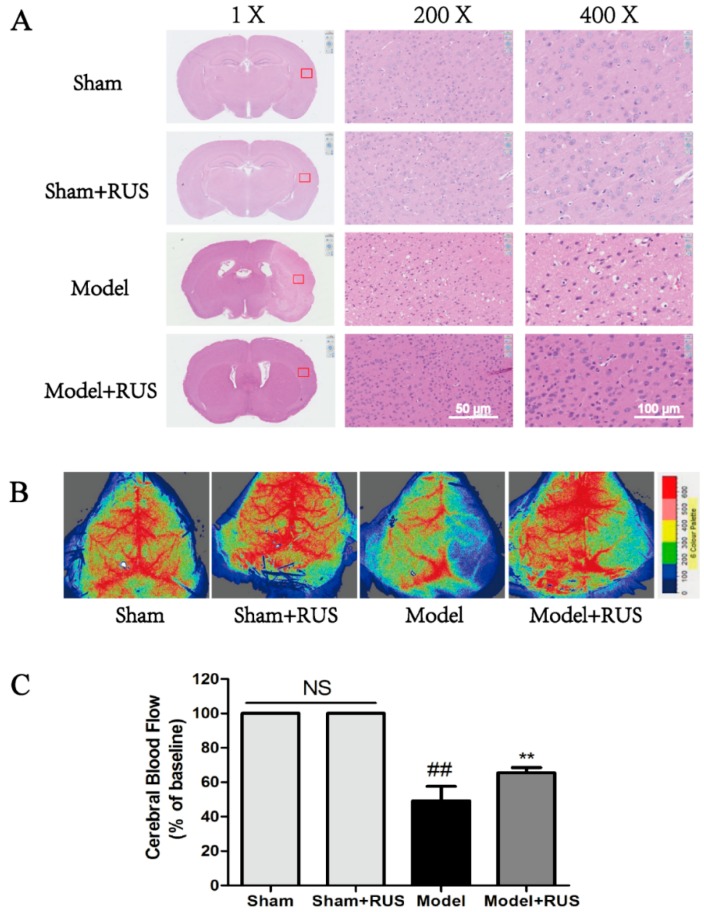
Effects of ruscogenin on histopathological changes and CBF in mice following MCAO/R. (**A**) Hematoxylin-and-eosin-stained slides of the brain sections of mouse in different groups; (**B**,**C**) representative images and quantitative analysis of cerebral blood flow of ipsilateral cortex in different groups. The magnitude of CBF is represented by different colors, with blue to red denoting low to high respectively (*n* = 6). NS means “not significant”. The data are expressed as means ± SD. ^##^ *p* < 0.01 vs. Sham, ** *p* < 0.01 vs. Model.

As Figure 2 shows, the hematoxylin and eosin (H & E) staining results demonstrated that the model group experienced neuronal loss and the presence of numerous vacuolated spaces; pretreatment with ruscogenin (10 mg/kg) could ameliorate histopathological damage by decreasing the cell loss compared with the model group (Figure 2A). Meanwhile, the quantification of cerebral blood flow (CBF) result showed that the administration of ruscogenin resulted in a significant increase in CBF compared with model group at 24 h reperfusion (Figure 2B,C). No obvious change was observed between Sham and Sham + RUS in the above experiments.

### 2.3. Ruscogenin Reduced the Evans Blue Leakage and Up-Regulates the Expression of Tight Junction Proteins Following MCAO/R

**Figure 3:**
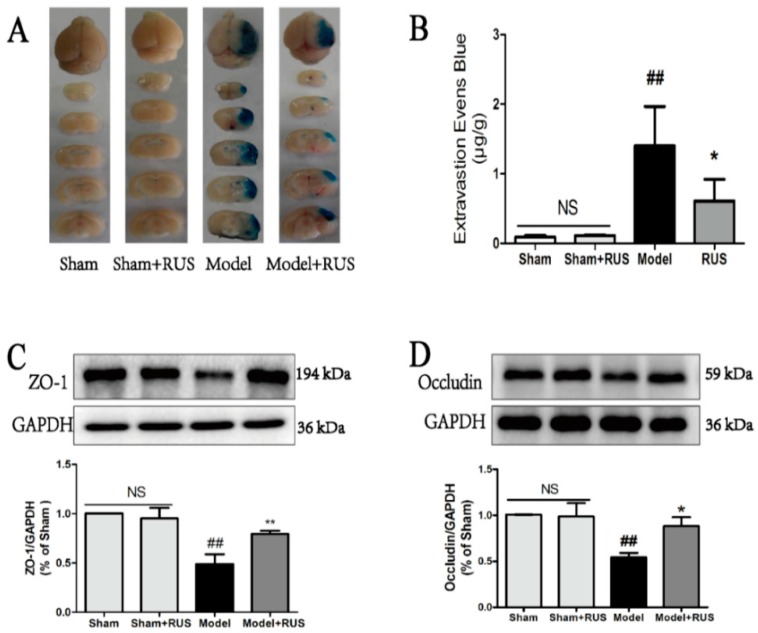
Effects of ruscogenin on BBB permeability and the expression of tight junction proteins in mice following MCAO/R. (**A**,**B**) Representative gross appearance of EB-stained brain section in mice and quantitative analysis of EB extravasation by spectrophotometry (*n* = 6); (**C**,**D**) representative Western blot bands and quantitative analysis of the expression levels of ZO-1 and occludin in different groups. The band intensities were measured using scanning densitometry. The data were normalized to glyceraldehyde-3-phosphate dehydrogenase (GAPDH) expression (*n* = 3). NS means “not significant”. The data are expressed as means ± SD. ^##^ *p* < 0.01 vs. Sham, * *p* < 0.05, ** *p* < 0.01 vs. Model.

BBB permeability was evaluated by the EB staining in the brains of mice, and the result of the quantitative spectrometry detected EB leakage in the model group and showed a significant EB extravasation compared with sham group and sham + RUS group at 24 h after reperfusion. Ruscogenin (10 mg/kg) treatment significantly reduced the leaked EB content (Figure 3A,B). We also investigated the effects of ruscogenin on the expression of ZO-1 and occludin at 24 h after reperfusion. Western blot analysis indicated that ruscogenin treatment could upregulate the decreased expressions of ZO-1 and occludin after ischemic stroke in mice (Figure 3C,D). No obvious change was observed between Sham and Sham + RUS in the above experiments.

### 2.4. Ruscogenin Inhibited the Expression of IL-1β and Caspase-1 and Modulated the TXNIP/NLRP3 Pathway Following MCAO/R

**Figure 4:**
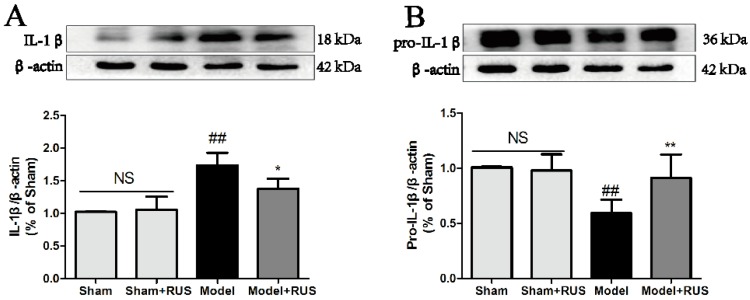

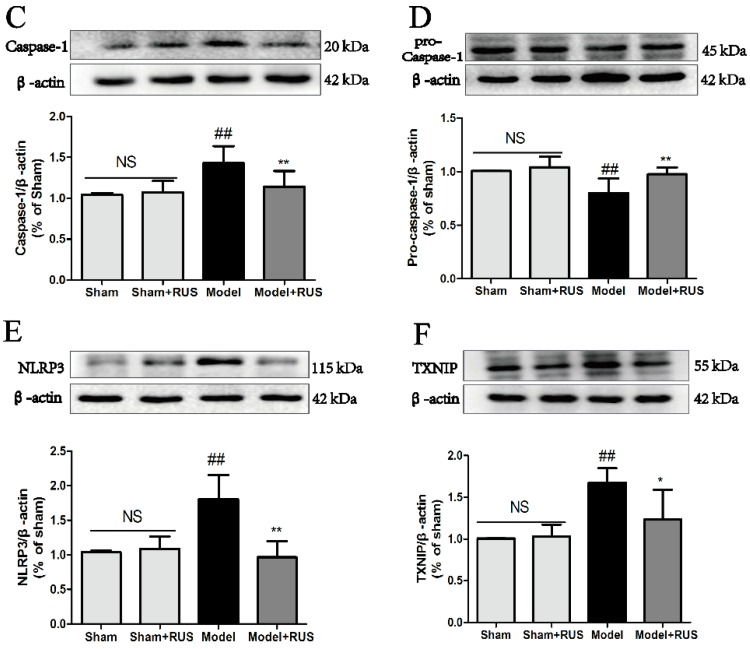
Effects of ruscogenin on the TXNIP/NLRP3 pathway in mice following MCAO/R. (**A**–**F**) Representative Western blot bands and quantitative analysis of the ratio of IL-1β and pro-IL-1β, caspase-1 and pro-caspase-1, NLRP3 and TXNIP. The band intensities were measured using scanning densitometry. The data were normalized to β-actin expression (*n* = 3). NS means “not significant”. The data are expressed as means ± SD. ^##^ *p* < 0.01 vs. Sham, * *p* < 0.05, ** *p* < 0.01 vs. Model.

To investigate the role of the TXNIP/NLRP3 pathway in MCAO/R-induced brain injury, we further investigated the expression of IL-1β, caspase-1, and the TXNIP/NLRP3 pathway at 24 h reperfusion. The Western blot results demonstrated that pretreatment with ruscogenin could inhibit the expression of IL-1β and caspase-1, and suppress the expression of NLRP3 and TXNIP (Figure 4A–F). No obvious change was observed between Sham and Sham + RUS in the above experiments.

### 2.5. Ruscogenin Increased the Cell Viability and Reverted the Barrier Leakage in bEnd.3 Cells Subjected to OGD/R

**Figure 5:**
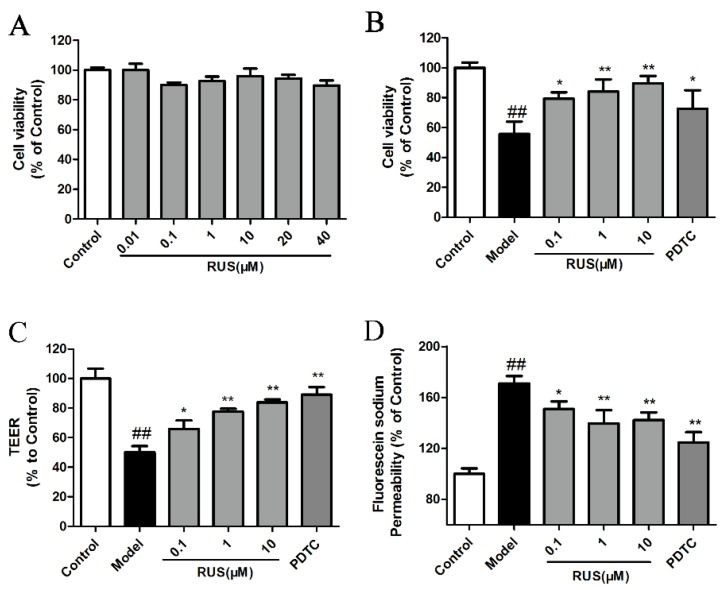
Effects of ruscogenin on the cell viability and barrier function in bEnd.3 cells subjected to OGD/R. (**A**,**B**) The bEnd.3 cells were treated with ruscogenin at various concentrations (0.01–40 µM) and the cell viability was measured using the 3-(4,5-dimethylthiazol-2-yl)-2,5-diphenyltetrazolium bromide (MTT) assay in normal conditions, or after 6 h of OGD and 18 h reoxygenation (*n* = 6); (**C**,**D**) the bEnd.3 cells were treated with ruscogenin (0.1–10 µM) and PDTC (10 µM), and subsequently exposed to 6 h of OGD and 18 h reoxygenation. The barrier-protection effect of ruscogenin was detected using TEER and sodium fluorescein assays. (*n* = 3). The data are expressed as means ± SD. ^##^ *p* < 0.01 vs. Control, * *p* < 0.05, ** *p* < 0.01 vs. Model.

Briefly, OGD/R was induced in a hypoxia chamber in RPMI 1640 culture medium without glucosein, in an atmosphere of 5% CO_2_, 94% N_2_ and 1% O_2_ for 6 h in the bEnd.3 cells, after which the cells were cultured under normoxia conditions for 18 h. We first investigated the various concentrations of ruscogenin on bEnd.3 cells under normoxia conditions in normal culture medium. The result suggested that pretreatment with ruscogenin (0.01–40 µM) had no obvious effect in normal bEnd.3 cells (Figure 5A). Then, we further investigated the effect of various concentration of ruscogenin (0.1–10 µM) pretreatment in bEnd.3 cells subjected to 6 h of OGD and 18 h reoxygenation. The results demonstrated that OGD/R-induced reduction of bEnd.3 cell viability was significantly recovered by the pretreatment with ruscogenin in various concentrations (Figure 5B). Meanwhile, the results of the trans-endothelial electrical resistance (TEER) value and fluorescein sodium permeability demonstrated that pretreatment with ruscogenin at various concentrations could increase the TEER value and inhibit the sodium fluorescein permeability compared with the model case (Figure 5C,D). Pyrrolidine dithiocarbamate (PDTC, 10 µM, a NF-κB inhibiter) also showed similar protection in bEnd.3 cells subjected to OGD/R (Figure 5B–D).

### 2.6. Ruscogenin Attenuated the Expression of Tight Junction Proteins and Actin Cytoskeleton Rearrangement in bEnd.3 Cells Subjected to OGD/R

**Figure 6:**
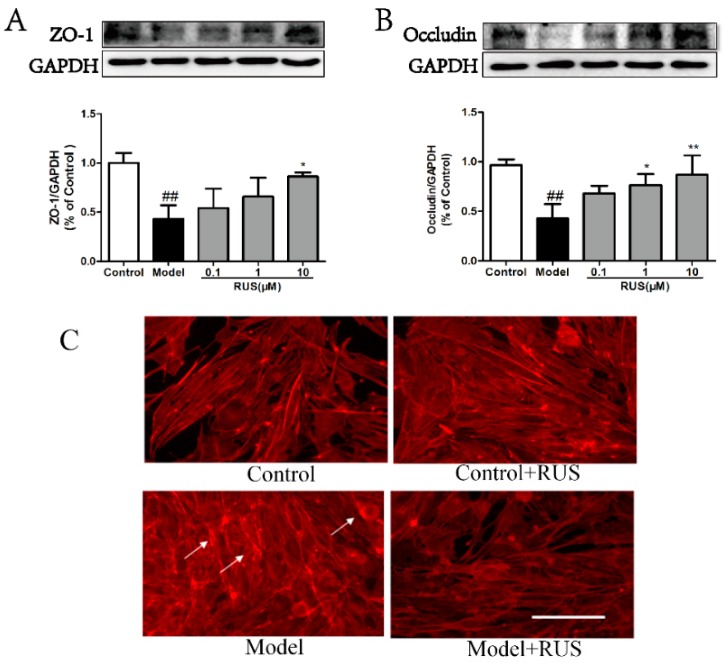
Effects of ruscogenin on the expression of tight junction proteins and actin cytoskeleton rearrangement in bEnd.3 cells subjected to OGD/R. (**A**,**B**) Representative Western blot bands and quantitative analysis of the expression levels of ZO-1 and occludin in different groups. The band intensities were measured using scanning densitometry. The data were normalized to GAPDH expression (*n* = 3). The data are expressed as means ± SD. ^##^ *p* < 0.01 vs. Control, * *p* < 0.05, ** *p* < 0.01 vs. Model; (**C**) representative immunostaining of F-actin in different groups in bEnd.3 cells subjected to OGD/R; the white arrow indicates the stress fiber. Scale bars = 100 µm.

To further identify the relationship between tight junction’s expression and BBB disruption, we detected the expression of ZO-1 and occludin using Western blot analysis. The Western blot results demonstrated that the expressions of ZO-1 and occludin were decreased in bEnd.3 cells subjected to 6 h of OGD and 18 h reoxygenation, whereas pretreatment with ruscogenin could recover these expressions in response to OGD/R in bEnd.3 cells (Figure 6A,B). Meanwhile, we also found that pretreatment with ruscogenin could ameliorate the OGD/R-induced endothelial cell stress fiber (white arrow) formation (Figure 6C).

### 2.7. Ruscogenin Inhibited the Expression of IL-Iβ and Caspase-1, and Modulated the TXNIP/NLRP3 Pathway in bEnd.3 Cells Subjected to OGD/R

**Figure 7:**
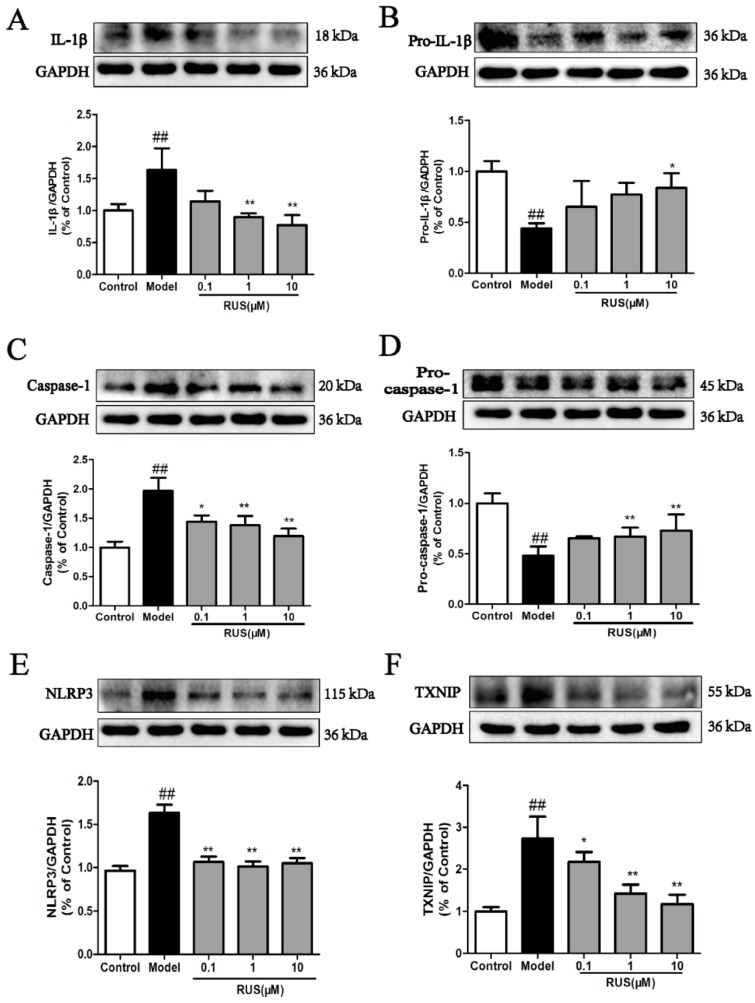
Effects of ruscogenin on the TXNIP/NLRP3 pathway in bEnd.3 cells subjected to OGD/R. (**A**–**F**) Representative Western blot bands and quantitative analysis of the ratio of IL-1β and pro-IL-1β, caspase-1 and pro-caspase-1, NLRP3 and TXNIP. The band intensities were measured using scanning densitometry. The data were normalized to GAPDH expression (*n* = 3). The data are expressed as means ± SD. ^##^ *p* < 0.01 vs. Control, * *p* < 0.05, ** *p* < 0.01 vs. Model.

We also investigated the expression of IL-1β, caspase-1 and the TXNIP/NLRP3 pathway in bEnd.3 cells subjected to 6 h of OGD and 18 h reoxygenation. The Western blot results demonstrated that pretreatment with ruscogenin could downregulate the increased expression of IL-1β and caspase-1 proteins, and inhibited the expressions of NLRP3 and TXNIP (Figure 7A–F).

### 2.8. Ruscogenin Inhibited the Production of ROS, and Regulated the MAPK Pathway in bEnd.3 Cells Subjected to OGD/R

**Figure 8:**
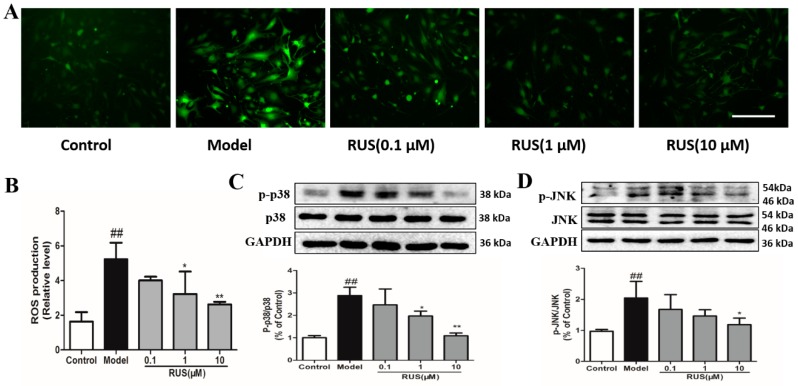
The effects of ruscogenin on ROS generation and the MAPK pathway in bEnd.3 cells after 6 h of OGD and 1h reoxygenation. (**A**) The intracellular ROS was viewed with fluorescence microscopy. Scale bar = 100 μm; (**B**) the resulting fluorescence intensity was quantified using ImageJ (National Institutes of Health, Bethseda, MD, USA); (**C**,**D**) representative Western blot bands and quantitative analysis of the ratio of p-p38/p38 and p-JNK/JNK; the band intensities were measured using scanning densitometry. The data were normalized to GAPDH expression (*n* = 3). The data are expressed as means ± SD. ^##^ *p* < 0.01 vs. Control, * *p* < 0.05, ** *p* < 0.01 vs. Model.

We further evaluated the effect of ruscogenin on ROS generation and the MAPK pathway after 6 h of OGD and 1 h of reperfusion in bEnd.3 cells. The results of fluorescence observations demonstrated that pretreatment with ruscogenin inhibited the production of ROS (Figure 8A,B), and the Western blot results indicated that ruscogenin could reduce p38MAPK and c-Jun N-terminal kinase (JNK) phosphorylation (Figure 8C,D).

## 3. Discussion

In the present study, we confirmed the efficacy of ruscogenin in MCAO/R-injured mice and OGD/R-injured bEnd.3 cells. In vivo results demonstrated that treatment with ruscogenin (10 mg/kg) could reduce brain infarction volume and edema and improve neurological deficits, histopathological damage, and CBF in MCAO/R-injured mice, and these effects might be related to its ability to ameliorate BBB breakdown and suppress TXNIP/NLRP3 inflammasome activation (Figure 1, Figure 2, Figure 3 and Figure 4). In vitro results correspondingly showed that ruscogenin (0.1, 1, 10 µM) could increase cell viability, decrease endothelial barrier leakage and upregulate the expression of tight junction proteins, modulate the TXNIP/NLRP3 inflammasome, and inhibit ROS generation and the MAPK pathway in the OGD/R-injured bEnd.3 cell (Figure 5, Figure 6, Figure 7 and Figure 8). All data revealed ruscogenin as a promising option for prevention of ischemic stroke. Meanwhile, the prevention of ischemic stroke has received attention due, to the increasing global stroke burden. Drugs for primary prevention of strokes, such as aspirin, are beneficial for human health [24,25]. Thus, our present study may provide an effective potential candidate drug for the prevention of ischemic stroke.

Ischemic stroke-induced brain damage is an extremely complex pathophysiological process, including multi-way, multifactor and multichannel damage [26,27]. However, current ischemic stroke research is largely focused on neurons and brain parenchyma, while direct protection of the BBB received much less attention. At the same time, abundant evidence has indicated that BBB dysfunction is a critical event during the progression of stroke, and that BBB integrity plays a key role in maintaining the microenvironment and homeostasis of the brain [3,28,29]. Thus, protecting the BBB from disruption may be a promising strategy for prevention and treatment of ischemic stroke. Moreover, it has been recognized that loss and degradation of ZO-1 and occludin could affect BBB integrity [29,30]. In the current study, our results suggested that ruscogenin treatment significantly reduced EB leakage in MCAO/R-injured mice and reversed endothelial barrier leakage in OGD/R-injured bEnd.3 cells, which indicates that ruscogenin could effectively improve BBB integrity (Figure 2 and Figure 5).

In addition, a number of studies have indicated that inflammation is a significant contributor to the pathological process of ischemic stroke [6,31,32], and that inflammation is a major cause of BBB disruption and leads to secondary tissue injury that increases the risk of bleeding and limits tissue recovery [11,33]. Thus, BBB permeability is closely linked to immune response and inflammatory response in the pathological process of ischemic stroke. In particular, innate immunity is the body’s first defense against pathogenic infection and recognizes pathogen-associated molecular patterns, possibly through pattern recognition receptors (PRRs) that activate downstream signaling pathways and provide immune response and inflammatory response [13,34]. Among these, inflammasomes are typical PRRs found in the pathology of ischemic stroke. They can be divided into three categories: NLRPs, NLR family CARD domain containing protein (NLRC), and neuronal apoptosis inhibitor protein (NAIP). Recent research has highlighted the key role of the NLRP3 inflammasome. NLRP3-mediated inflammatory response is involved in atherosclerosis and the development of ischemic stroke, and regulation of NLRP3 inflammasome activation may play a critical role in the prevention and treatment of ischemic stroke [35,36,37]. The NLRP3 inflammasome can activate caspase-1 and serve as a key mediator and a platform for maturation of IL-1β and other cytokines [38,39]. Furthermore, TXNIP, as the endogenous inhibitor and regulator of thioredoxin, which could rapidly bind to NLRP3 inflammasome, and triggering assembly and oligomerization of the inflammasome [16,40,41]. Moreover, previous reports have demonstrated that TXNIP expression is upregulated in brain tissue after stroke in animals, and ROS induces the dissociation of TXNIP and actives NLRP3 [41,42]. Thus, inhibiting ROS generation and modulating the TXNIP/NLRP3 pathway might be beneficial for prevention and treatment of ischemic stroke. In our present study, we found that ruscogenin could inhibit the expression of caspase-1 and IL-1β, and suppress TXNIP/NLRP3 inflammasome activation in MCAO/R-injured mice and OGD/R-injured bEnd.3 cells (Figure 4 and Figure 7). Moreover, we also verified that ruscogenin could reduce the production of ROS and inhibit the MAPK pathway (Figure 8). All these findings demonstrated that ruscogenin ameliorated BBB disruption and might be related with suppressing the TXNIP/NLRP3 and MAPK pathways. Current studies on the inhibition of the inflammasome activation by saponins is extremely limited, thus our findings well enrich their potential application in related diseases.

Aside from these findings, several potential limitations of this present study exist, due to other possible underlying mechanisms in the ischemic stroke-induced BBB dysfunction, including the phosphatidylinositide 3-kinases/protein kinase B (PI3K/Akt), NF-κB and rho-associated coiled coil-containing protein kinase-1/myosin light chain (ROCK1/MLC) signaling pathways [43,44,45]. Whether or not ruscogenin could protect the BBB disruption through these or other related signaling pathways remains unclear. In this present study, our data demonstrated that ruscogenin could inhibit the expression of caspase-1 and IL-1β, and modulate the TXNIP/NLRP3 pathway in vivo and in vitro. As previous reports have described [23], ruscogenin could also ameliorate the cerebral ischemia-induced brain damage and inflammatory responses via inhibition of the NF-κB signaling pathway. It implies that ruscogenin protects BBB breakdown following ischemic stroke and might be related to the NF-κB signaling pathway. In addition, as an anti-inflammatory agent, ruscogenin could suppress mouse neutrophil activation through the Akt, MAPK, and protein kinase A (PKA) pathways [22]. Therefore, whether or not ruscogenin protects the BBB dysfunction through these signaling pathways is of great interest for further investigation.

## 4. Materials and Methods

### 4.1. Ethics Statement

These experiments were carried out in accordance with the National Institutes of Health Guide for the care and use of laboratory animals (NIH Publication No. 80-23, revised 1996). All procedures and assessments were approved by the Animal Ethics Committee of China Pharmaceutical University (Permission NO. 20150306).

### 4.2. Reagents

Ruscogenin was isolated from the tubers of *Ophiopogon japonicus* and the purity of the sample obtained analyzed using high performance liquid chromatography-evaporative light scattering detection (HPLC-ELSD) was 98.6% by successive chromatographic steps [23]. MTT and PDTC were purchased from Ameresco (Solon, OH, USA). TTC, fluorescein sodium and Evans blue (EB) were purchased from the Sigma-Aldrich Company Ltd. (St. Louis, MO, USA).

### 4.3. Animals and Treatment

Male C57BL/6J mice weighing 18–22 g (8–10 weeks old) were purchased from the Model Animal Research Centre of Yangzhou University (Yangzhou, China, certificate No. SCXK 2014-0004). Mice were housed in a temperature-controlled environment with a 12-h-light-dark cycle and allowed free access to food and water. Prior to experiments, animals were randomized into different experimental groups and the indices were measured blindly.

### 4.4. Focal Cerebral Ischemia

The mice for the experimental stroke model were induced by MCAO and reperfusion in C57BL/6J mice as reported previously [46]. Briefly, animals were anesthetized with 4% chloral hydrate (0.1 mL/10 g body weight) intraperitoneally (i.p.), then the neck vessels were exposed with a midline incision, and branches of the right external carotid artery were carefully isolated and cauterized. Next, a 6–0 nylon monofilament suture, blunted with silicon-coated tip was advanced 9–10 mm into the internal carotid to occlude the origin of the MCA. The body temperature of animals were maintained with a heating pad (Alcbio, Shanghai, China) at 37.0 ± 0.5 °C during surgery and ischemia. Meanwhile, sham-operations were carried out with the same procedure, except that the suture was not advanced into the internal carotid artery. Using a laser Doppler flow meter (LDF; FLPI2, Moor, UK) to confirm the decrease of the middle cerebral artery blood flow immediately after the occlusion to below 30% of the basic cerebral blood flow [47]. About 1 h after occlusion, the suture was withdrawn to allow reperfusion for 24 h.

### 4.5. Cell Culture

bEnd.3 cells were obtained from the Bioleaf Biotech Co., Ltd. (Shanghai, China). Cell were cultured in RPMI 1640 (Invitrogen, Carlsbad, CA, USA) and supplemented with 15% fetal bovine serum (FBS, Sigma, St. Louis, MO, USA), 100 U/mL penicillin, and 100 U/mL streptomycin (Ameresco, Columbus, OH, USA) at 37 °C in a humidified atmosphere of 5% CO_2_ and 95% air. The growth medium was changed every day and cells were plated onto 96-well plates or petri dishes at a density appropriate to be used in further experiments.

### 4.6. Oxygen-Glucose Deprivation and Drug Treatment

Ruscogenin and PDTC were dissolved in RPMI 1640 culture medium, without glucose, at various concentrations (0.1, 1, and 10 μM) to adjust the final Dimethyl Sulfoxide (DMSO, SunshineBio, Nanjing, China) concentration to 0.1% (*v*/*v*). After the bEnd.3 cells were treated, OGD/R was induced in the cells for 6 h in a hypoxia chamber in RPMI 1640 culture medium without glucosein, in an atmosphere of 5% CO_2_, 94% N_2_ and 1% O_2_, following which, the cells were cultured under normoxia conditions.

### 4.7. Evaluation of Infarct Volume, Neurological Deficits, and Cerebral Water Content

To further confirm whether ruscogenin exerts a protective effect in mice, the mice were randomly divided into four groups: Sham, Sham + RUS (ruscogenin), Model, and Model + RUS, Ruscogenin was dissolved in 0.5% sodium carboxymethyl cellulose at a dose of 10 mg/kg. Mice were given ruscogenin or equal volume of 0.5% sodium carboxymethyl cellulose by intragastric administration for 1 h before MCAO. After 1 h MCAO and 24 h of reperfusion, mice were anesthetized with 4% choral hydrate (i.p.), the brains were quickly removed, and then TTC staining was performed to measure the brain infarction. Data are expressed as a percentage of total hemispheres [23]. Brain water contents were determined 24 h after reperfusion and using the wet-dry method, as previously described [48]. Neurological deficits of the experimental animals were evaluated according to Longa’s method, as previously described [46]. Briefly, the measurement of neurological scores were tested as follows: 0: no deficit; 1: forelimb weakness and torso turning to the ipsilateral side when held by the tail; 2: circling to the affected side when held by the tail on the bench; 3: unable to bear weight on the affected side or spontaneous circling to the affected side; 4: no spontaneous locomotor activity or barrel rolling.

### 4.8. Histomorphological Analysis and Cerebral Blood Flow

Histomorphological analysis was measured by hematoxylin-eosin (H & E) staining, as reported previously [49]. The histomorphological analysis was conducted by a pathologist blinded to the treatment groups. CBF was measured by a laser Doppler flowmetry as described previously [50]. The images were acquired at 24 h after reperfusion in mice.

### 4.9. Evaluation of BBB Permeability

As described previously [51], BBB permeability was evaluated by the leakage of EB into the brain via tail vein injection. Briefly, 2 h before the animals were euthanized, 0.1 mL/10 g body weight 2% EB in normal saline was injected into every animal. Then, the ipsilateral hemisphere was removed and homogenized in 1mL of trichloroacetic acid and centrifuged at 12,000× *g* for 20 minutes. Supernatants were collected and quantitatively determined by measuring the 620 nm absorbance with a spectrophotometer.

### 4.10. Cell Viability and Trans-Endothelial Electrical Resistance Assay

Cell viability was measured through MTT assays, as previously described [45]. The integrity of the bEnd.3 cell monolayer was measured via the TEER assay using a Millicell ERS-Volt-Ohm Meter (Millipore, Billarica, MA, USA). As reported previously [45], the cell seeding density was about 5 × 10^4^ in 200 μL of complete RPMI 1640 media. The TEER values are shown as Ω × cm^2^ based on the culture inserts.

### 4.11. Measurement of Fluorescein Sodium Permeability

Endothelial barrier leakage was detected using sodium fluorescein across a bEnd.3 cell monolayer, as previously described [52]. Briefly, the Millicell suspension culture chamber transwell inserts (Millipore) were placed in 24-well plates. Paracellular permeability was evaluated by the addition of Krebs-Ringer buffer (KRB; 118 mM NaCl, 4.7 mM KCl, 1.3 mM CaCl_2_, 1.2 mM MgCl_2_, 1.0 mM NaH_2_PO_4_, 25 mM NaHCO_3_, and 11 mM d-Glucose, pH = 7.4) containing 100 μg/mL sodium fluorescein to the top chamber. The concentration of sodium fluorescein was determined after 30 min at 37 °C using a fluorescence multiwall plate reader (Thermo Fisher, Waltham, MA, USA) with a reference wavelength of 485 nm and 530 nm.

### 4.12. Detection of ROS Production

Intracellular ROS generation was measured by using 2′,7′-dichlorodihydrofluorescein diacetate (DCFH-DA, Beyotime, Shanghai, China) as a fluorescent probe. As previously described [53], after treatment the bEnd.3 cells were incubated with DCFH-DA (1 μΜ) for 30 min at 37 °C in the dark. The dye was excited at 488 nm, and the emission was detected at 525 nm by fluorescence microscope (Leica, Mannheim, Germany). Images were quantified by fluorescence intensity using ImageJ (National Institutes of Health, Bethseda, Rockville, MD, USA).

### 4.13. Western Blot Analysis

Western blotting analysis was performed as reported previously [54]. In brief, the brain tissues or cells were lysed and centrifuged at 12,000× *g* for 10 min at 4 °C. Equal amounts of proteins (30 μg) were loaded into 10%–12.5% SDS-PAGE and transferred to PVDF membranes (Millipore Corporation, Billerica, MA, USA) by electrophoresis. After blocking with 5% BSA for 1.5 h, samples were incubated overnight at 4 °C with primary antibodies against ZO-1 (1:200, Abcam, Cambridge, MA, USA), occludin (1:200, Abcam), caspase-1 (1:500, Abcam), IL-1β (1:500, Abcam), TXNIP (1:200, Santa Cruz Biotechnology, Dallas, TX, USA), NLRP3 (1:200, Santa Cruz Biotechnology), p38 and phospho-p38 (1:1000, CST, Boston, MA, USA), JNK and phospho-JNK (1:1000, CST),GAPDH (1:8000, Bioworld, Louis Park, MN, USA) or β-actin (1:2000, Bioworld). The blots were then incubated with horseradish peroxidase (HRP)-conjugated anti-rabbit or anti-mouse secondary antibody (dilution 1:8000, Bioworld) and developed with enhanced chemiluminescence (ECL, Vazyme Biotech, Nanjing, China). The immune-reactive bands were visualized using the ChemiDoc™ MP System (Bio-Rad, Hercules, CA, USA) and the results were quantified by using the Image Lab^TM^ Software (version 4.1, Bio-Rad, Hercules, CA, USA).

### 4.14. Immunofluoresence Analysis

To investigate the actin cytoskeleton in OGD/R-injured bEnd.3 cells, as previously described [55], the cells were incubated with rhodamine-labelled phalloidin dye (diluted into 1:400, Sigma) at room temperature for 60 min. The fluorescent images were visualized with a Leica fluorescence microscope (Leica, Wetzlar, Germany).

### 4.15. Statistical Analysis

Statistical analysis was carried out using Student’s two-tailed t test for comparison between two groups, and one-way analysis of variance (ANOVA) followed by Dunnett’s test when the data involved three or more groups. Results are expressed as mean ± SD. *p* < 0.05 was considered statistically significant. All analyses were performed with GraphPad Prism Version 5.01 (GraphPad Software Inc., San Diego, CA, USA).

## 5. Conclusions

**Figure 9:**
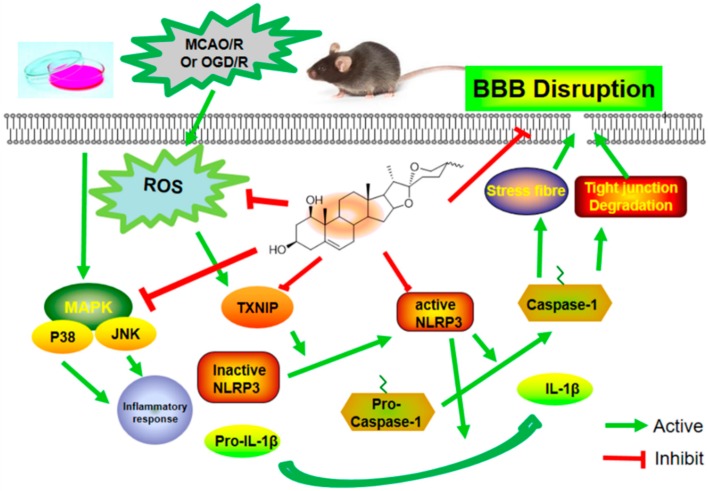
Proposed mechanisms of ruscogenin on BBB dysfunction after ischemic stroke. Upon hypoxia and ischemia, ROS is generated and MAPK activated; activated TXNIP activates the inactivated NLRP3 inflammasome, and triggers the expression of IL-1β and caspase-1, which contribute to the decreased tight junctions’ expression, stress fiber formation and changes in cell permeability. Ruscogenin ameliorates the ischemia-hypoxia-induced BBB disruption through upregulating the expression of tight junction proteins, and suppressing the expression of IL-1β and caspase-1, modulating the TXNIP/NLRP3 inflammasome activation and MAPK pathway.

In conclusion, our present study further verifies the protective role of ruscogenin in MCAO/R-injured mice, and provides in vitro pharmacological evidence using OGD/R-injured bEnd.3 cells. In addition, our data also suggest that ruscogenin can protect against MCAO/R-induced BBB permeability and OGD/R-induced endothelial barrier function. Further studies found that the underlying mechanisms might be related to suppressing TXNIP/NLRP3 inflammasome activation (Figure 9). These findings might provide new insights and pharmacological evidence of its potential application for the prevention and treatment of ischemic stroke.

## Abbreviations

BBB	Blood-brain barrier
CBF	Cerebral blood flow
EB	Evans Blue
IL-1β	Interleukin-1β
MAPK	Mitogen-activated protein kinase
NLRP3	Nucleotide-binding domain (NOD)-like receptor family, pyrin domain containing 3
PKA	Protein kinase A
PDTC	Pyrrolidinedithiocarbamate
PRRs	Pattern recognition receptors
ROS	Reactive oxygen species
TJs	Tight junctions
TXNIP	Thiredoxin-interactive protein
ZO-1	Zonula occludens-1
